# The Ninth International Medical Congress

**Published:** 1885-11

**Authors:** 


THE NINTH INTERNATIONAL MEDICAL CONGRESS.

Notwithstanding the "spleenful mutiny" on the part of the *Medical News*, the *New York Medical Journal* and the *New York Medical Record*, the prospects of a successful INTERNATIONAL MEDICAL CONGRESS, at Washington, in 1887, continue to be encouraging. The professional eminence, energy and perseverance of the medical gentlemen, to whom the preliminary arrangements of the Congress have been intrusted, render a favorable issue of this vexed question highly probable. The *animus* of the conduct of the New York Medical Journals in the controversy is obvious. Commercial interests have unfortunately substituted just regard for professional honor. We hesitate to ascribe to personal pique the anomalous conduct of the *Medical News.* Yet the scrupulous regard to probity which has characterized the first century's existence of the publishing house—of which the *News* is the organ—forbids any other conclusion.

